# Psychological Distress in Healthy Low-Risk First-Time Mothers during the Postpartum Period: An Exploratory Study

**DOI:** 10.1155/2017/8415083

**Published:** 2017-01-16

**Authors:** Christina Murphey, Patricia Carter, Larry R. Price, Jane Dimmitt Champion, Francine Nichols

**Affiliations:** ^1^School of Nursing, The University of Texas at Austin, Austin, TX, USA; ^2^College of Education & Department of Mathematics, Texas State University, San Marcos, TX, USA; ^3^Maternal-Child Research Consultants, Austin, TX, USA

## Abstract

Psychological distress, defined as depression, anxiety, and insomnia in this study, can occur following the birth of a baby as new mothers, in addition to marked physiological changes, are faced with adapting to new roles and responsibilities. We investigated the cooccurrence of stress, depression, anxiety, and insomnia in mothers during the postpartum period; tested the feasibility of study methods and procedures for use in this population; and identified new mothers interest in using cranial electrotherapy stimulation (CES) as an intervention for reducing psychological distress. We recruited healthy, low-risk, English speaking first-time mothers, ages 18–32 years, with healthy babies (*N* = 33), within 12 months of an uncomplicated birth. Participants completed the PSS, HAM-D_14_, HAM-A_17_, and PSQI_19_. No problems were encountered with study procedures. Mothers reported a high interest (4.9) in the potential use of CES to treat or prevent the occurrence of psychological distress. All participants (*N* = 33) reported moderate levels of depression and anxiety, while 75.8% (*n* = 25) reported insomnia. PSS scores were within the norms for healthy women. Further research is recommended to investigate if our findings can be replicated or if different patterns of associations emerge. Implications for clinical practice are addressed.

## 1. Introduction

The birth of a child is a major life event [1] that can be filled with excitement, anticipation, and joy. However, the transition and adaptation to new demands, roles, responsibilities, and changes in relationships can be stressful, especially for first-time mothers. In addition, new mothers typically encounter physiological changes and struggle with concerns about weight gain, body image, sexuality, and other physical difficulties such as fatigue [2]. These problems may generate or exacerbate stress and lead to an actual or perceived crisis [3] and psychological distress.

Psychological distress, defined as depression, anxiety, and insomnia in this study, often increases during the postpartum period [4] and can negatively affect maternal mental health status, maternal and family functioning, and infant-child outcomes. Depression, anxiety, and insomnia commonly present as comorbidities [5–7] but are often unrecognized in clinical practice [8] or undertreated as comorbidities in new mothers [9]. This unrecognized cluster of comorbidities may lead to psychological distress and subsequently poor outcomes for mothers, their infants, and children [10].

Current treatment recommendations for depression, anxiety, and insomnia are primarily pharmaceutical or psychotherapy, both of which have limitations related to cost, time involved, and ineffectiveness for some women. Consequently, there is a need to examine other treatment approaches including complementary modalities, such as cranial electrotherapy stimulation (CES), particularly considering current evidence that shows the efficacy of early detection, intervention, and treatment for pregnant and postpartum women [11].

The goals of this exploratory study are consistent with developmental work and preparation needed for a future complex intervention study [12]. The aims were to (1) investigate the relationships among stress, depression, anxiety, and insomnia in healthy low-risk first-time mothers during the postpartum period, (2) test the feasibility, suitability, and acceptability of study methods and procedures for use in this population, (3) determine new mothers' interest in using the Alpha-Stim® AID CES device as an intervention for reducing depression, anxiety, and insomnia, and (4) test the psychometric properties of the Perceived Stress Scales.

## 2. Background

New mothers experience numerous psychological and physiological changes after the birth of a child. The most common psychological problems in new mothers during the postpartum period are depression, anxiety, and insomnia. The interrelationships among depression, anxiety, and insomnia experienced by mothers during the postpartum period are well documented [13–16]. In a large depression screening study of 10,000 women during the postpartum period Wisner and colleagues [16] reported that, of the 566 women who screened positive for depression, almost two-thirds (*n* = 374; 66.1%) had a secondary diagnosis of anxiety disorder. In the 24 women who had a primary diagnosis of anxiety disorder, 96% (*n* = 23) had a secondary diagnosis of depression disorder.

There is a bidirectional, additive relationship between insomnia and depression. Insomnia is an established risk factor for depression in new mothers, while research shows that depression can lead to insomnia [14]. The comorbidity diagnosis is known to increase symptomatology in the primary diagnosis [15]. During pregnancy and the postpartum period, women who have more clinically significant (more severe) insomnia are also likely to have depression and generalized anxiety [15].

### 2.1. Depression

Depression is one of the most debilitating disorders for childbearing women [17] and is considered an international public health problem [18] with potential life-course implications for maternal and infant-child health [19, 20]. The term “postpartum depression” describes depression that persists or occurs after the tenth postpartum day and may extend to the first postpartum year [2, 21]. Depression during pregnancy and the postpartum period is more widely studied than stress, anxiety, or insomnia.

Although reported incidence and prevalence rates differ [22], it is estimated that depression, depressive symptoms, and associated mood disorders may affect between 8 and 29% of childbearing-aged women in the United States (US) [23–27]. Others have reported the occurrence to be as high as 30% [28, 29]. Women who report depression and depressive symptoms have higher rates of long-term adverse infant and child outcomes including developmental and cognitive impairments, low self-esteem, and self-regulation and temperament difficulties [15, 30–32].

Factors related to perinatal and postpartum depressive symptoms are well established and include a history of depression, perceived poor health, alcohol and cigarette use, unemployment, low socioeconomic status, young maternal age, being unmarried [33], and ethnicity minority status [34]. Finally, studies have shown that depressive symptoms are associated with levels of stress and number of stressful life events [35–37] and that pregnant and postpartum women may experience more stressful events than nonpregnant women [38] or nonpostpartum women.

### 2.2. Anxiety

Although the incidence and clinical course of postpartum depression have been well established [17], there is limited research on postpartum anxiety. Determining the epidemiology of postpartum anxiety is problematic due to contextual factors such as family and social background and maternal health status during the peripartum period [39]. Rates of postpartum anxiety are thought to be greatly underestimated and may range within 20–25% [40–42]. Because depression and anxiety are frequent comorbidities, it is likely that postpartum women who report depressive symptoms also experience clinical symptoms of anxiety [5, 43]. However, international professional clinical guidelines for postpartum depression do not generally call for the inclusion of assessment and screening of anxiety [44].

Anxiety disorders without the comorbidity of depression are particularly common in childbearing women [45], and the onset of many anxiety disorders is in early adulthood [46], a time when many women are considering childbirth and motherhood [43]. Research indicates that childbirth is a stressor [9] and that postpartum anxiety is a common experience among women of childbearing age [43, 47]. Past studies suggest that a considerable portion of women experience anxiety during the perinatal and postpartum period [22]. Further, prolonged exposure to maternal depression and anxiety is related to adverse psychological and behavioral problems in children [48, 49].

### 2.3. Insomnia

New mothers commonly report insomnia, reduced sleep duration, and poor sleep quality. A longitudinal study of the effects of pregnancy on mother's sleep conducted by Hedman and colleagues [50] reported that sleep characteristics changed from the first trimester (increased deep sleep), through the second and third trimesters (progressively less total sleep), and the poorest sleep quality was reported during the first three months postpartum. Complaints of insomnia are common from delivery to three months postpartum [51]. Another study conducted by Insana, Statcom, and Mongomery-Downes [52] explored both objective and subjective sleep quality in new mothers during the first several months postpartum and found that participants consistently reported fragmented sleep that was correlated with increased levels of daytime fatigue, mood disturbance, and reduced psychomotor vigilance.

Therefore, it is important not to dismiss sleep problems in new mothers as a “normal” part of transition into motherhood, as chronic sleep deprivation can result in insomnia. In turn, insomnia has been linked to increased depressive symptoms [6, 53, 54]. These findings further support the negative impacts that maternal insomnia may have on maternal health and infant-child development and safety.

### 2.4. Barriers to Standard Care and Treatment

Standard care and treatment is standardized around high-level evidence and thus represents the best current therapy [55] that new mothers receive. Only about half of women with depression and anxiety are identified; and even fewer receive adequate treatment [56]. Once diagnosed, a woman with depression, anxiety, and insomnia may receive standard care that includes pharmacotherapy (i.e., antidepressant medication) specific types of psychotherapy, such as cognitive behavioral therapy, or both. In general, treatment choices depend on the type of disorder, the woman's preference, and the expertise of the clinician [57, 58]. Although pharmacotherapy and psychotherapy can be effective medical management modalities, researchers have identified several barriers to this standard care for mothers seeking help for depression, anxiety, and insomnia.

Personal and access barriers include the ability to differentiate between symptoms that are self-manageable and those symptoms that warrant professional help [59–61], reluctance to seek help due to shame, stigma related to an inability to cope, being viewed by society as a “bad mother” [59–61], a preference for self-management [59, 60], time and infant-child care constraints [62], the inability to access a clinician [63], and rejection of standard care and cost. Turner and colleagues [63] reported specific barriers to pharmacotherapy and psychotherapy in their randomized control trial (RCT) of 27 women in three UK cities. Researchers identified main barriers to antidepressant medication, fear that symptoms were being masked by the antidepressants and delaying recovery, lack of access to a health care provider, and concerns related to medication dependency and side effects. Some participants reported taking a lower dose of medication than prescribed as a result of these concerns. Women were also concerned that antidepressant medication might affect their ability to function as a mother in terms of sleepiness and breastfeeding. A barrier to psychotherapy was the belief of the women that they could talk to their peers and therefore professional counseling was not warranted.

### 2.5. Cranial Electrotherapy Stimulation

Standard care and treatment for depression, anxiety, and insomnia have side effects (i.e., pharmacotherapy), limited availability (i.e., psychotherapy), and other barriers that reduce their use and effectiveness. Clearly, there is a need for additional treatment approaches. In this study we sought to determine new mothers' interest in using CES as a treatment for psychological distress during the postpartum period. Based on the research, CES has the potential to decrease depression, anxiety, and insomnia of mothers during the postpartum period. While positive anecdotal clinical reports are available, no research was found on mothers' use of CES during the postpartum period.

Obstacles to using CES should be fewer than the barriers to standard care and treatment of depression, anxiety, and insomnia. The Alpha-Stim AID CES device is safe, readily available, easy to use, and suitable for clinical or home use. One potential barrier could be cost. However, the Alpha-Stim AID is cost effective compared with standard care and treatment and many insurance plans that cover durable medical equipment will pay for Alpha-Stim technology [64]. Thus, the benefits of using the Alpha-Stim AID could potentially outweigh any potential barriers to use for new mothers during the postpartum period.

**Figure 1:**
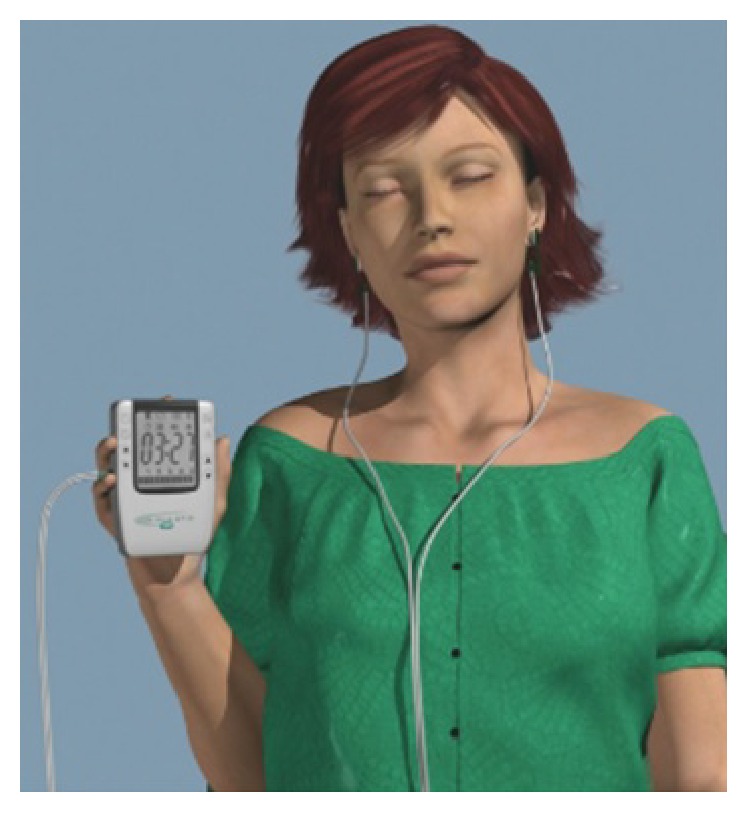
Alpha-Stim CES device.

About the size of a smart phone, the Alpha-Stim AID CES device (see Figure 1) delivers a mild electrical current to the brain using ear clip electrodes; the current creates a state of alpha brainwaves which is associated with relaxation [64]. The most common side effects are headache, dizziness, and irritation at the electrode sites, and these are mild and occur in less than 1% of individuals. Headache and dizziness are related to the current being set a too high a level for an individual. Both symptoms decrease and resolve when the current is decreased. Irritation at the electrode sites can be managed by changing the placement of the electrodes. CES was cleared by the US Food and Drug Administration for the treatment of depression, anxiety, and insomnia in 1979 [64]. In Europe, the Alpha-Stim AID CES device is a Class IIa, Type BF medical device. Research findings to date, using the Alpha-Stim AID CES device, indicate that CES is effective for the treatment of depression, anxiety, and insomnia in adults [65, 66]. In the US, a prescription is required to purchase a CES device. However, outside of the US and worldwide, the Alpha-Stim AID CES device is available without a prescription [64].

## 3. Materials and Methods

### 3.1. Design and Sample Size

An exploratory, cross-sectional design was used to investigate the study aims. The study was approved by the university Institutional Review Board. Informed written consent was obtained from all participants. Effect sizes were not available to determine sample size. Because this study was designed to explore possible associations between depression, anxiety, and insomnia in first-time mothers, test the feasibility, suitability, and acceptability of study methods and procedures, for use in this population, and identify new mothers' interest in using CES for psychological distress, a sample size of 33 (30 plus 10% for attrition) was determined to be acceptable using Browne's rule [67].

### 3.2. Participants

This nonprobability purposive sample was recruited from two community health care clinics and one private obstetrical clinic located in the Southwestern US. Eligible women were healthy low-risk first-time mothers within 12 months of delivery who were scheduled for postdelivery follow-up care or well-baby care visits with a primary health care provider from three recruitment locations. New mothers were included if they were within 18–40 years of age, who gave birth to a healthy infant within 12 months of recruitment, read and spoke English, and could give informed consent.

Exclusion criteria included women who had complications during or after delivery requiring admission to an adult intensive care unit or whose infants were admitted to neonatal intensive care unit (NICU), a multiple birth (e.g., twins, triplets) or stillbirth, women who had a history of diagnosed mental health or chronic physical health condition that was not well controlled, or women currently pregnant via self-report. Of the 42 women assessed for eligibility, nine (21%) were excluded, three (7%) were under 18 years of age, three (7%) had ongoing infant complications that required the infant's admission to the NICU, and three (7%) were lost to follow-up. Some individuals had multiple exclusion criteria. There were 33 participants enrolled in this study.

### 3.3. Recruitment, Screening, and Enrollment

New mothers were provided information about the study by clinic staff. If the individual indicated interest in participating in the study, she received a study invitation letter and screening packet from the clinic staff. After reading the study invitation letter, the new mother was asked to complete the background and demographic form (screening packet) and the PSS_4_ a brief stress inventory. The PSS_4_ is often used clinically and in research to identify maternal psychological stress [68]. The data were used to determine preliminary eligibility for participation. The principal investigator (PI) reviewed the background and demographic form and screened the participant's responses against the inclusion and exclusion criteria. The potential participant was notified if she qualified for the study or did not meet the criteria for the study. If a new mother qualified for the study, the PI briefly described the study and time commitment. If the new mother agreed to participate, an appointment was scheduled at the participants' next scheduled clinic visit with the PI to complete four questionnaires (PSS_10_, HAM-D_17_, HAM-A_14_, and PSQI_19_) and participate in an in-person audio-recorded interview.

The PI explained the study's purpose and procedures and read the consent form to all potential participants. After the potential participant's questions were addressed and verbal consent to participate was obtained, the new mother signed the informed consent form. The participant completed the data collection process in a private area in the clinic. The procedure for administration of the questionnaire applied to all instruments (PSS_10_, HAM-D_17_, HAM-A_14_, and PSQI_19_). The PI read each question verbatim to the participant as they followed along with a copy of the questionnaire. The PI rated and recorded the answer for each questionnaire item based on the participants' response.

Following the completion of questionnaires, the new mother participated in a discussion with the PI and answered interview questions. During this interview, the PI described and demonstrated how the Alpha-Stim AID is self-applied and used. The PI used an inactive model of the Alpha-Stim AID for the demonstration. The participant was given a handout detailing the Alpha-Stim AID to follow along during the PI's self-application demonstration. Following the demonstration, the PI asked the participant questions about their perceptions of the usefulness of the Alpha-Stim AID as a potential treatment for depression, anxiety, and sleep disturbance in new mothers. Data collection time for both study components (questionnaires and interview) was approximately 60 minutes' total per participant. All activities were completed in one visit.

### 3.4. Instruments

A total of seven instruments were used to measure study variables. Two instruments were investigator-developed forms to elicit subject background data. Five clinical instruments were used to measure outcomes.

#### 3.4.1. Demographic and Background Form

An investigator-developed, self-report form was used to describe demographic characteristics, current lifestyle behaviors, health and obstetric history, and infant-feeding method.

#### 3.4.2. Semistructured Interview Guide

A semistructured interview guide was developed for this study to elicit participants' responses about the acceptability of Alpha-Stim AID CES therapy as an intervention for reducing depression, anxiety, and insomnia. Items for this measure were anchored on a five-point Likert-type scale ranging from 1 (never true) to 5 (very often true). Open ended sample questions related to CES are presented as follows.

*Sample Participant Open-Ended Questions about Alpha-Stim AID CES*

*Sample Questions*

- If you could design a device or activity that would help you with symptoms of stress (depression, anxiety, and insomnia) what would that look like?
- After hearing about the Alpha-Stim AID, what are your thoughts and opinions about how useful this device would be for new mothers experiencing depression, anxiety, and insomnia?

#### 3.4.3. Perceived Stress Scales

The Perceived Stress Scale_4_ (PSS_4_) and the Perceived Stress Scale_10_ (PSS_10_) were selected as they are widely used during the postpartum period to measure psychological stress.

#### 3.4.4. Depression, Anxiety, and Insomnia

Three standardized clinical scales, each measured depression, anxiety, and insomnia, were selected because they are commonly used in mental health practice to evaluate the presence of depression (HAM-D_17_), anxiety (HAM-A_14_), and insomnia (PSQI_19_). A description of the clinical instruments is depicted as follows.

*Summary of Study Instruments*

*PSS*_*4*_. The Perceived Stress Scale_4_ (PSS_4_): The PSS_4_ is widely used as a clinical screening instrument for measuring the perception of stress. During the postpartum period the scale_4_ is often used as a screening measure to determine psychological stress. However, in a critical review of the literature, no research could be found where the findings from the PSS_4_ are compared with the findings from standardized clinical scales for anxiety, depression, and insomnia. The PSS_4_ was used in this study to evaluate its usefulness as a screening scale for psychological distress. The PSS_4_ is a 4-item self-report instrument with a five-point scale (0 = never, 1 = almost never, 2 = sometimes, 3 = fairly often, and 4 = very often) appropriate for use in situations requiring a very brief measure of stress perceptions [69]. The PSS_4_ is based on accepted psychometric principles and is considered to be valid based on its correlation with other depression scales. The Cronbach's alpha for a sample of overweight, low-income postpartum women was 0.80 [70]. It is not a diagnostic instrument. No cut-off scores have been established for the scale. Scores acquired on the PSS_4_ were used to compare scores between samples using descriptive statistics [71].

*PSS*_*10*_. The Perceived Stress Scale_10_ (PSS_10_) is the most widely used psychological instrument for measuring the perception of stress. In the postpartum period it is often used as a screening measure to determine psychological stress. However, in a critical review of the literature, no research could be found where the findings from the PSS_10_ are compared with the findings from standardized clinical scales for anxiety, depression, and insomnia. It measures the degree to which a person perceives life as stressful during the past month [72]. It is not a diagnostic instrument. No cut-off scores have been established for the scale and provides only norms for comparisons between samples [71]. It measures the degree to which a person perceives life as stressful during the past month [72]. It is not a diagnostic instrument. No cut-off scores have been established for the scale and provides only norms for comparisons between samples [71]. The score range is 0 to 56 with higher scores indicating higher levels of stress. The higher degree and longer duration of self-perceived stress, indicated by a higher score, are considered a risk factor for a clinical mental health disorder [71]. The scale has established validity and reliability with internal consistency values between 0.85 and 0.87. Questions are of a general nature and widely used in general and perinatal populations [73].

*HAM-D*_*17*_. The Hamilton Depression Rating Scale_17_ (HAM-D_17_) is a 17-item comprehensive observer scale administered by a health care professional or researcher that assesses the type and magnitude of symptom burden present and is therefore considered to be a measure of illness severity [74]. It is used widely in psychopharmacology trials and has established reliability and validity in the literature. Zimmerman et al. (2013) in a study of 627 outpatients with major depressive disorder (MDD) established the following cut-off severity scores: no depression (0–7); mild depression (8–16); moderate depression (17–23); and severe depression (≥24) [75, 76].

*HAM-A*_*14*_. Hamilton Anxiety Rating Scale_17_ (HAM-D_14_): The HAM-A_14_ is an observer scale designed to quantify the severity of anxiety symptoms and to assess the response to therapeutic interventions [77]. It is a 14-item instrument that measures current anxiety symptoms. The scales' items measure anxious mood, insomnia, depressed mood, tension, fears, intellectual impairment, somatic muscular and sensory complaints, cardiovascular, respiratory, and gastrointestinal symptoms, genitourinary symptoms, autonomic symptoms, and patient's affect at interview [77] The optimal HAM-A_14_ score ranges include no/minimal anxiety ≤7; mild anxiety = 8–14; moderate = 15–23; severe ≥ 24 [78]. The instrument exhibits established reliability and validity in the literature [79]. The investigator who conducted the interviews was trained in the administration of the HAM-A_14_.

*PSQI*_*19*_. Pittsburg Sleep Quality Index (PSQI_19_): The PSQI_19_ is a 19-item scale that measures sleep quality during the previous month and discriminates between good and poor sleepers [80]. It has established validity in the literature and can be used to identify insomnia symptoms. The scale has internal consistency and a reliability coefficient of 0.83. A PSQI_19_ global score > 5 (≥6) resulted in a sensitivity of 98.7 and specificity of 84.4 as a marker for sleep disturbances in insomnia patients versus controls [81].

### 3.5. Data Analysis

The IBM Statistical Package for the Social Sciences (SPSS), version 23.0, was used for data analyses. All survey data were entered directly into the SPSS software program. Effect size analyses were conducted in addition to statistical tests of significance for the bivariate correlation analyses. Given the exploratory nature of this investigation and the marginal sample size, effect size analysis served to provide information on the magnitude and direction of association and to document effects for sample size planning in a future study of larger scale [82].

## 4. Results

### 4.1. Descriptive Statistics

The participants in this study were English speaking healthy, low-risk, first-time mothers (*N* = 33) within 12 months of an uncomplicated childbirth between the ages of 18 and 32 inclusive (M = 22.3, SD = 4.57). Twenty-one percent were White (*n* = 7), 9% were Black/African American (*n* = 3), 64% were Hispanic/Latina (*n* = 21), and 6% reported as being of more than one ethnicity. The demographic characteristics of new mothers in this study are shown in Table 1.

### 4.2. Cranial Electrotherapy Stimulation

The third aim of our study is an aim of special interest as the next step in this program of research is to determine the efficacy of the Alpha-Stim AID CES therapy as an intervention reducing depression, anxiety, and insomnia among first-time new mothers during postpartum period in a future RCT. The participants responded affirmatively to the information about using CES for depression, anxiety, and insomnia and the demonstration of the Alpha-Stim AID. They indicated that the potential use of CES for the treatment of psychological distress was both suitable and acceptable for new mothers during the postpartum period. The total score from the semi structured interview guide on new mothers' interest in using CES for psychological distress was 4.9 on the five-point Likert-type scale ranging from 1 (never true) to 5 (very often true). Sample comments from participants included “*Can I try it now?”*;* “Great idea, no medications!*”;* “I can do it by myself and at home”*; and, “*Good for ‘me' time that will help me feel better*.” Their questions focused on the ability for the device to maintain an electrical charge without interrupting treatment delivery. They expressed a desire to meet in their home or another public location for subsequent study visits for a future study.

### 4.3. Reliability of Instruments

In our study, Cronbach's alphas for the PSS_4_ and PSS_10_ items were 0.42 and 0.82, respectively. The coefficient alphas for the HAM-A_14_ instrument to measure anxiety and HAM-D_17_ to measure depression were 0.52 and 0.32, respectively. The Cronbach alpha for the PSQI was 0.72.

### 4.4. Stress, Depression, Anxiety, and Insomnia

A comparison of participants' scores from the PSS_4_ (M = 5.36; SD = 2.1) and PSS_10_ (M = 17.39; SD = 5.9) with their scores from the HAM-D_17_, HAM-A_14_, and PSQI_19_ revealed that the PSS_4_ and PSS_10_ were both significantly moderately correlated with depression, anxiety, and insomnia in this study (see Table 1).

**Figure 2:**
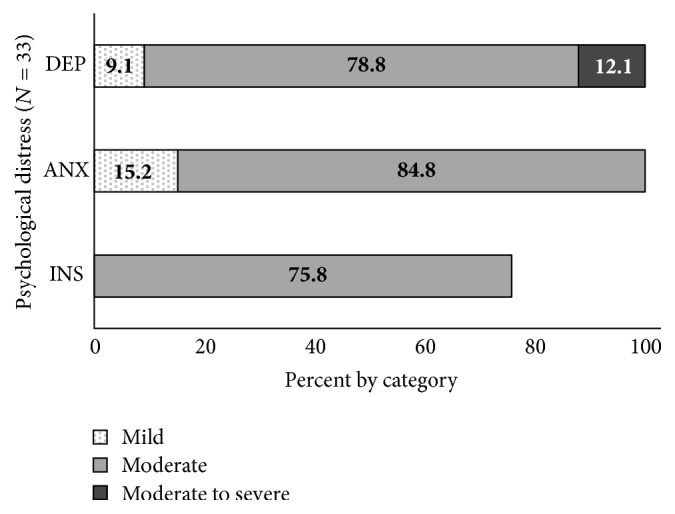
Level of depression, anxiety, and insomnia by category. DEP = depression; ANX = anxiety; INS = insomnia.

Findings from the HAM-D_17_, HAM-A_14_, and PSQI_19_ revealed that all participants (*N* = 33) displayed scores indicative of depression and anxiety, while 25 (75.8%) of the participants scored in the moderate range for insomnia. The levels of depression, anxiety, and insomnia by category are shown in Figure 2.

### 4.5. Relationships between Stress, Depression, Anxiety, and Insomnia

Table 2 provides the M, SD, Mdn, and range for instruments.  Table 3 depicts results of the bivariate correlation analysis for the measures used in this study. Nine statistically significant Pearson correlation coefficients were observed among the outcome variables for the participants.

However, post hoc power analysis based on the sample size of *N* = 33, Type I error rate of 0.001 (corrected for the number of correlation coefficients tested), and 0.53 average correlations revealed a power of 0.55. The low level of statistical power in turn yielded an unacceptable Type II error rate. Alternatively, effect size analyses yielded a medium effect range for the nine correlation coefficients [82].

## 5. Discussion

### 5.1. Participants

The women who participated in this study were healthy, low-risk, first-time mothers with healthy babies who had not sought treatment for depression, anxiety, and insomnia during the postpartum period and who were not taking any medications for these disorders. The characteristics of participants (see Table 1) were representative of the women receiving postpartum care at the recruitment sites.

### 5.2. Recommendation for Future CES Studies

Recommendations for future studies are to explore efficacy of the CES and other targeted interventions to improve symptoms of psychological distress in a pilot study that includes a larger sample of first-time new mothers. Women in our study expressed a desire for self-management of depression, anxiety, and insomnia and the desire to meet at alternative locations from their primary care services. CES provides a self-management of symptoms of psychological distress and has the potential to decrease barriers to standard of care and treatment for future studies with this population. Recruitment strategies to include adolescent women are recommended. Three healthy low-risk adolescent women were excluded because they were <18 years old. Adolescent women are a vulnerable and underserved group and should be included in future studies because CES technology is safe for adolescent women and may be technologically appealing.

### 5.3. Stress

Findings from the screening PSS_4_ revealed that participants in our study (*N* = 33) perceived higher stress (M = 5.36; SD = 2.1) than in a descriptive study (*N* = 168; M = 4.81; SD = 3.03) of behavioral and psychosocial health in an ethnically and economically diverse sample of postpartum women [70]. A review of the raw data from our study revealed that if the PSS_4_ had been used as a screening scale to determine entry into this study, the cut-off score would need to be ≥8. Requiring this score on the PSS_4_ would have eliminated 29 of the 33 participants in the study whose scores on the HAM-D_17_ and HAM-A_14_ indicated depression and anxiety, respectively. This cut-off score would have also eliminated all 21 participants whose scores on the PSQI_19_ indicated insomnia symptoms. Thus, in this study, the PSS_4_ did not function well as a screening instrument for psychological distress (depression, anxiety, and insomnia).

Participants in our study had higher stress scores on the PSS_10_ than in two studies of women in a US general population in 2006 and 2009 [83] and lower stress scores than in three studies. One study included overweight low-income postpartum women in the US [84], while two studies of Arabic speaking healthy postpartum women in Lebanon included participants from four different populations in 2010 [85] and 2014 [86]. See Table 4 for comparison of our findings with previous studies.

Consistent with the PSS_4_, the PSS_10_ was not a good predictor of depression and anxiety in this study. Using a cut-off score of 20 for psychological distress, very reasonable and possibly too low, 20 out of 33 participants in this study scored below 20 on the PSS_10_ (no or minimal stress), but all scored in the mild or moderate range for depression and anxiety on the HAM-D_14_ and HAM-A_17_, respectively.

One limitation of using the PSS is that only normative scores are available in the literature. Cut-off scores to classify levels of stress such as mild, moderate, or severe have not been established [71]. During interviews conducted in our investigation, it was evident that participating women were experiencing “stress” during the postpartum period as expressed by their responses and comments. Additionally, scores on the HAM-D_17_, HAM-A_14_, and PSQI_19_ confirmed this observation. Yet the PSS_4_ and PSS_10_ scores indicated that only four out of 33 women were experiencing anxiety and depression and none of the women experiencing insomnia exhibited “stress.” One explanation for this observation is that the PSS_4_ and PSS_10_ measure different dimensions of stress than the HAM-D_17_, HAM-A_14_, and PSQI_19_.

### 5.4. Depression, Anxiety, and Insomnia

Our study demonstrates that healthy, low-risk, first-time mothers experienced depression, anxiety, and insomnia during the postpartum period. One potential explanation for the high occurrence of depression, anxiety, and insomnia relates to the issue of self-selection into the study. For example, mothers who enrolled in this study were interested in joining the study because of the psychological distress they were experiencing. The comorbidities of depression, anxiety, and insomnia using the HAM-D_17_ and HAM-A_14_ and PSQI_19_ were high among participating women. The HAM-D_17_, HAM-A_14_, and PSQI_19_ scales used in this study captured the psychological distress that these new mothers were experiencing. Our literature review revealed no instances where the HAM-D_17_ and HAM-A_14_ clinical scales were used with “healthy,” low-risk, first-time mothers with healthy infants. The mothers in our study may reflect the two-thirds of women who are depressed and have secondary diagnosis of anxiety [16] and are unrecognized in clinical practice [8] or undertreated [9]. Therefore, it may be time for a shift in thinking about maternal stress and psychological distress as a cluster of comorbidities rather than focusing on individual diagnoses of depression, anxiety, and insomnia [87, 88].

### 5.5. Limitations

The main limitations of this exploratory study include the cross-sectional design and small sample size. An additional limitation is that new mothers did not use CES in this study; they indicated an interest in using CES. However, we believe it was important to know if women would be interested in the technology prior to designing and implementing a complex intervention study. Regardless of these limitations, the results from this study raise important questions for future research, particularly related to findings of depression, anxiety, and insomnia in a healthy “well” sample of first-time mothers. The findings here are valuable for screening and assessment in clinical practice where the primary focus is typically physical recovery during the postpartum period rather than psychological distress in first-time mothers.

## 6. Conclusion

Further research is recommended to investigate if our findings can be replicated or if different patterns of associations emerge. Critical to future research is a larger sample size and more rigorous research design. Importantly, the findings highlight a need for mental health screening and a broader approach to thinking about maternal stress and psychological distress in this population. Moreover, based on participants' positive comments regarding the nonpharmacological, noninvasive, and self-management aspect of the proposed intervention, clearly the mothers in our study were interested in using CES to treat or prevent the occurrence of the depression, anxiety, and insomnia. The results of this exploratory study support a set of feasible and acceptable data collection procedures and outcome measures suitable [89] for studying psychological distress in first-time new mothers during the postpartum period. The knowledge gained from this study is critical in terms of achieving methodological rigor and efficient implementation [90] for a future RCT using the Alpha-Stim AID.

## Figures and Tables

**Table 1:** Demographic characteristics of participants (*N* = 33).

Characteristic	*n*	%
Marital status		
married	11	33
single, father of baby not involved	10	30
single, father of baby involved	12	37
Living arrangement		
living with spouse or father of baby	17	52
living with family	16	48
Number of people living with you (excluding self)		
1–5	25	76
6–10	8	24
Income		
0–19,000	18	55
20,000–39,999	8	24
40,000–69,999	4	12
70,000–90,999	1	3
100,000–150,000	2	6
Education		
Did not complete high school	7	21
High school graduate (or GED)	14	43
Some college credit	5	15
Associate degree	2	6
Bachelor's degree	4	12
Master's degree	1	3
Currently employed		
Yes	9	27
No	25	72
How do you pay for healthcare?		
Medicaid	24	73
Private pay	6	18
Missing	3	9
Type of pregnancy		
Singleton	33	100
Type of delivery		
Vaginal birth	25	76
Cesarean birth	8	24
Current infant feeding method		
Breast milk only	6	18
Formula only	15	46
Breast milk and formula	12	36

**Table 2:** M, SD, Mdn, and range for instruments (*N* = 33).

Instrument	Mean	SD	Mdn	range
PSS_4_	05.36	2.104	05	1–10
PSS_10_	17.39	5.900	18	4–25
HAM-A_14_	21.55	2.916	22	16–27
HAM-D_17_	20.15	2.682	20	13–23
PSQI_19_	08.91	3.189	10	1–16

PSS_4_ = 4-item Perceived Stress Scale; PSS_10_ = 10-item Perceived Stress Scale; HAM-A_14_ = Hamilton Anxiety Scale, 14-item; HAM-D_17_ = Hamilton Depression Scale, 17-item; PSQI_19_ = Pittsburg Sleep Quality Scale, 19-item.

**Table 3:** Pearson correlation coefficients for selected outcome measures (*N* = 33).

	1	2	3	4	5
(1) HAM-D_17_	1	0.60^*∗∗*^	0.54^*∗∗*^	0.60^*∗∗*^	0.57^*∗∗*^
(2) HAM-A_14_	—	1	0.28	0.46^*∗∗*^	0.57^*∗∗*^
(3) PSQI_19_	—	—	1	0.41^*∗*^	0.51^*∗∗*^
(4) PSS_4_	—	—	—	1	0.51^*∗∗*^
(5) PSS_10_	—	—	—	—	1

*Note*. ^*∗*^ *p* < 0.05; ^*∗∗*^ *p* < 0.01. HAM-D_17_ = Hamilton Depression Scale, 17-item; HAM-A_14_ = Hamilton Anxiety Scale, 14-item; PSQI_19_ = Pittsburg Sleep Quality Scale, 19-item; PSS_4_ = 4-item Perceived Stress Scale; PSS_10_ = 10-item Perceived Stress Scale.

**Table 4:** Comparison of PSS_10_ mean scores of new mothers in this study (M = 17.39, SD = 5.9) to PSS_10_ mean scores reported in the literature.

Study	*N* and sample	PSS_10_ scores
Walker et al., (2012)	*N* = 71	Scores from the Walker et al. were *higher* than in our study
	Overweight, low-income postpartum women	(i) White/Anglo (M = 20.3, SD = 6.0)
	(i) White/Anglo (*n* = 23)	(ii) African American (M = 21.8, SD = 5.7)
	(ii) African American (*n* = 25)	(iii) Hispanic (M = 20.0, SD = 5.7)
	(iii) Hispanic (*n* = 23)	

Osman et al., (2014)	*N* = 123	Scores from the Osman et al. study were *higher *than in our study
	(i) Healthy first-time mothers in Beirut, Lebanon	M = 18.93, SD = 7.03

Cohen and Janicki-Deverts, (2012)	*N* = 2066	Scores from the Cohen and Janicki-Deverts study were *lower* than in our study
	Healthy women in a general population of females in the US	(i) 2006 (M = 16.10, SD = 7.30)
	(i) 2006 (*n* = 1034)	(ii) 2009 (M = 16.4, SD = 7.56)
	(ii) 2009 (*n* = 1032)	

Chaaya et al., (2010)	*N* = 268	Scores from the Chaaya et al. study were *higher* than in our study
	(i) Pregnant women (*n *= 113)	(i) Pregnant women (M = 18.0, SD = 5.7)
	(ii) Postpartum women (*n *= 97)	(ii) Postpartum women (M = 18.3, SD = 4.8)
	(iii) Healthy, university students, nongravid, nonmothers (*n *= 58)	(iii) University women (M = 20.3, SD = 4.8)
	(iv) Arabic speaking women in Beirut, Lebanon	
